# The bovine foot skin microbiota is associated with host genotype and the development of infectious digital dermatitis lesions

**DOI:** 10.1186/s40168-022-01440-7

**Published:** 2023-01-10

**Authors:** V. Bay, A. Gillespie, E. Ganda, N. J. Evans, S. D. Carter, L. Lenzi, A. Lucaci, S. Haldenby, M. Barden, B. E. Griffiths, E. Sánchez-Molano, R. Bicalho, G. Banos, A. Darby, G. Oikonomou

**Affiliations:** 1 Institute of Infection, Veterinary and Ecological Sciences, University of Liverpool, Liverpool, UK; 2 Faculty of Agriculture, Ege University, İzmir, Turkey; 3 Department of Animal Science, Penn State University, State College, PA USA; 4 The Roslin Institute, University of Edinburgh, Edinburgh, UK; 5 FERA Diagnostics and Biologicals, College Station, TX USA; 6 Scotland’s Rural College (SRUC), Easter Bush, Midlothian, UK

## Abstract

**Background:**

Bovine Digital Dermatitis (BDD) is a prevalent infectious disease, causing painful foot skin lesions and lameness in cattle. We describe herein the bovine foot skin microbiota and its associations with BDD using 16S rRNA gene amplicon and shotgun metagenomic sequencing on samples from 259 dairy cows from three UK dairy farms.

**Results:**

We show evidence of dysbiosis, and differences in taxonomy and functional profiles in the bovine foot skin microbiome of clinically healthy animals that subsequently develop BDD lesions, compared to those that do not. Our results suggest that taxonomical and functional differences together with alterations in ecological interactions between bacteria in the normal foot skin microbiome may predispose an animal to develop BDD lesions. Using genome-wide association and regional heritability mapping approaches, we provide first evidence for interactions between host genotype and certain members of the foot skin microbiota. We show the existence of significant genetic variation in the relative abundance of *Treponema* spp. and *Peptoclostridium* spp. and identify regions in the bovine genome that explain a significant proportion of this variation.

**Conclusions:**

Collectively this work shows early changes in taxonomic and functional profiles of the bovine foot-skin microbiota in clinically healthy animals which are associated with subsequent development of BDD and could be relevant to prevention of disease. The description of host genetic control of members of the foot skin microbiota, combined with the association of the latter with BDD development offer new insights into a complex relationship that can be exploited in selective breeding programmes.

Video Abstract

**Supplementary Information:**

The online version contains supplementary material available at 10.1186/s40168-022-01440-7.

## Introduction

Bovine Digital Dermatitis (BDD) is a prevalent infectious disease, causing painful foot skin lesions. This results in cattle becoming lame which in turn compromises animal welfare and causes significant production losses [1]. Many BDD-associated pathogens are also considered commensals of the foot skin, gastrointestinal tract and faeces of ruminants, or ubiquitous to the farm environment. The polymicrobial nature of this disease has led to the hypothesis that the foot skin microbiota, and the relationships between its members, may affect occurrence and progression of lesions [2].

Changes in bacterial populations in bovine foot skin throughout the progression of BDD lesions have been investigated using both 16S rRNA gene and shotgun metagenomic sequencing [1, 3–7]. Analysis from lesion biopsies showed that temporal changes in the foot skin microbiota composition and diversity occur at each of the five morphologically distinct lesion stages. *Treponema* species, which are the most common pathogens associated with BDD, were found in low abundance in early lesions and became dominant in latter stage lesions [3, 6]. However, the foot skin microbiota of clinically healthy cows is yet to be investigated in terms of its potential role in future disease development [2]. Functional differences in motility/chemotaxis, respiration, iron acquisition, phosphorus metabolism, cell division and cell cycle, and regulation and cell signalling, have been demonstrated between healthy and diseased foot skin microbiomes; however, it is unknown if these differences are detectable prior to the appearance of visible BDD lesions [3].

Links between host genetics and the abundance of certain taxa in the microbiome have been made in several settings and our understanding of how host genotype affects the microbiome is evolving. Mechanisms of host genetic control of the microbiome have been discussed in the context of the human gut microbiome. A genetic variant may directly cause a particular phenotype, with alterations to the microbiome being a consequence of disease. Alternatively, different genotypes may alter gene expression, which will affect the microbiome; or a genetic variant may directly alter the microbiome resulting in disease [8]. Srinivas et al. [9] investigated the contribution of host genetics to the skin microbiota using a fourth generation of an advanced intercross mouse line with 1,199 informative SNPs. They demonstrated 3 significant and 6 suggestive quantitative trait loci (QTL) associated with 9 operational taxonomic units (OTUs). Using the 15^th^ generation of the same mouse line and increasing the single nucleotide polymorphism (SNP) number to 53,203, Belheouane et al. [10] also investigated the effect of host genetics on skin microbiota, describing 21 significant SNP-skin microbiota associations and identifying genes related to skin inflammation and cancer. However, the association of the bovine foot skin microbiota and BDD related bacteria with the host genetics remains unclear.

We describe herein the bovine foot skin microbiota using 16S rRNA amplicon and shotgun metagenomic sequencing on skin swab samples taken from dairy cows from three UK dairy farms. We show differences in the foot skin microbiome profiles of clinically healthy animals that were associated with subsequent development of BDD. We also present the first co-occurrence analysis of the bovine foot skin microbiome showing ecological relationships among bacterial species. We hypothesise that taxonomical and functional differences, and differences in ecological interactions between bacteria in the normal foot skin microbiome may predispose an animal to development of BDD lesions. Additionally, using genome-wide association and regional heritability mapping approaches, we provide first evidence for significant interactions between host genotype and certain members of the bovine foot skin microbiota.

## Materials and methods

### Detailed description of methodology is provided in the Supplementary Information

#### Ethics and overview of the study population

Ethical approval for the study was granted by University of Liverpool Research Ethics Committee. Procedures regulated by the Animals (Scientific Procedures) Act (ASPA) were conducted under a Home Office Project License (Reference Number: PPL 70/8330).

### Sample collection and classification into foot-health groups

Primiparous and multiparous Holstein cows (259) from three farms (detailed description of the three farms is provided by Griffiths et al. 2020 [11]) were enrolled between October 2016 and June 2017 and approximately 3–4 weeks prior to their expected calving. Animals were restrained in a handling crush suitable for lifting feet for inspection. This study used skin surface swabs to sample the foot skin so that cows could be followed longitudinally and inspected for development of BDD lesions without invasive skin biopsies disrupting the susceptible area which would have artificially increased the risk of infection. To collect samples, the back-left foot was lifted, and gross contamination removed using a clean paper towel. Sterile cotton swabs were used to sample the area of the foot most susceptible to developing BDD lesions, namely at the skin-horn junction of the heel bulbs [12]. Samples were initially kept on ice and were frozen at -80 °C within a few hours for use in 16S rRNA gene sequencing. Although samples were collected only from the back- left foot (for reasons associated with project logistics), all four feet were inspected, and lesions recorded of any of the five clinical BDD stages according to the established M-scoring system [13]. Feet were inspected on three further occasions: one week, four weeks and 8–10 weeks post-calving. This resulted in classification of the study population into four foot-health groups based on foot lesion data for all four feet: Healthy/Healthy (HtHt, *n* = 112) cows never had digital dermatitis on any foot, Healthy/Infected (HtIn, *n* = 48) were healthy on all feet pre-calving, but subsequently developed BDD (observed at any of the five clinical stages at one or more of the three inspections and on any of the inspected feet), Infected/Infected (InIn, *n* = 59) had BDD (on any of the inspected feet) pre-calving and remained infected throughout the study, and Infected/Healthy (InHt, *n* = 16) had BDD pre-calving (on any of the inspected feet) but recovered by the second inspection. Shotgun metagenomic analysis was undertaken for five samples from each of the HtHt and HtIn groups (all from the same farm) to compare cows that developed BDD with those that did not with higher taxonomic resolution and to investigate differences in functional profiles. Certain analyses were also performed for the following groups of animals: BL_HtHt (*n* = 148): cows which had healthy back left feet during the study, BL_HtIn: cows which were healthy at sampling, then developed BDD on their back left feet, BL_InIn: cows which had BDD on their back left feet throughout the study, BL_InHt: cows which had BDD on their back left feet pre-calving and then recovered.

**Fig. 1:**
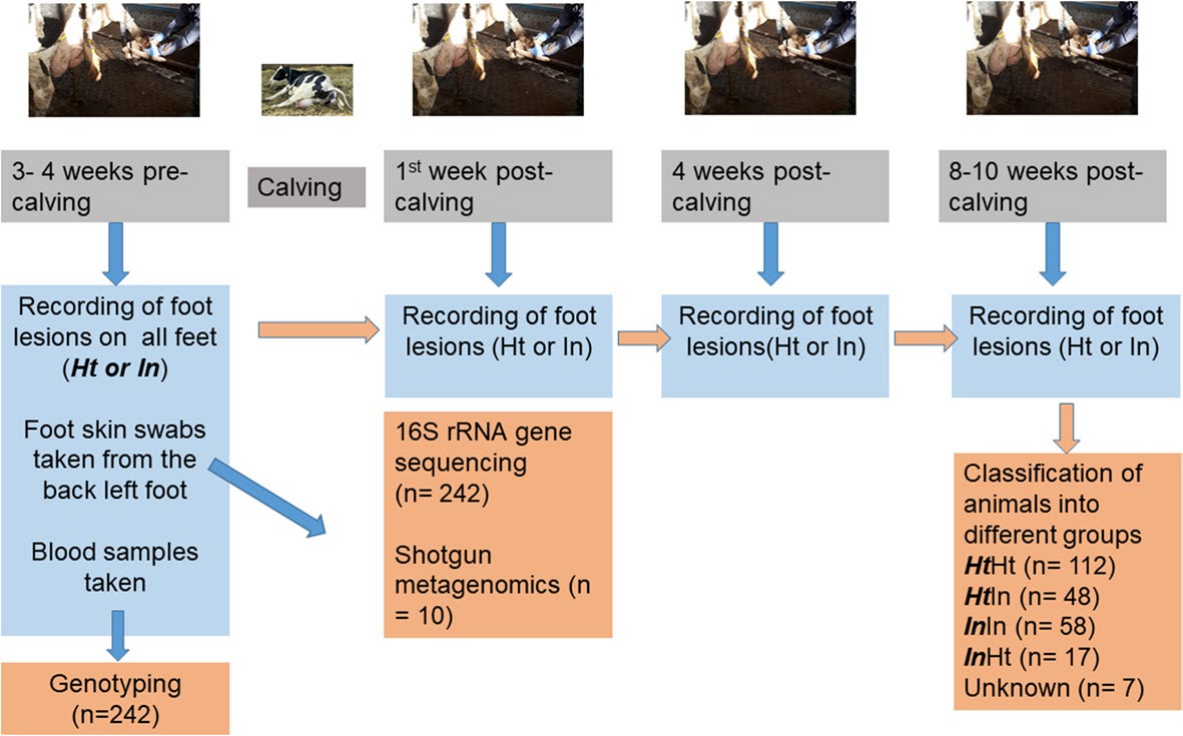
Timeline of sampling illustrating the process for classification of cows into foot-health groups, and numbers of samples used for 16S rRNA sequencing, shotgun metagenomics sequencing and GWAS

In a genome-wide association study (GWAS), 554 cows from the three farms were studied to identify genomic regions and potential candidate genes associated with lameness traits [14]. This also allowed us to investigate associations between host genotype and the foot skin microbiota for 242 of these animals for which both foot skin microbiome and genomic data were available. Figure 1 illustrates the sampling process and number of samples used for 16S rRNA sequencing, shotgun metagenomics sequencing and GWAS.

### DNA extraction, 16S rRNA gene amplification, and sequencing

Microbial DNA was extracted from collected swabs using the PureLink™ Microbiome DNA Kit (Invitrogen, Carlsbad, CA, USA) which utilizes chemical, heat and bead-beating cell lysis prior to purification. Extracted DNA samples were stored at -20 °C until amplification for sequencing. DNA was also extracted from two swabs that were opened on farm but were not used to sample cows; these served as negative controls. Amplification of the V3-V4 hypervariable region of the 16S rRNA gene for sequencing was conducted using Illumina_16S_341F and Illumina_16S_805R universal primers with adapter sequences [15]. Amplicons were sequenced using the Illumina® HiSeq 2500 platform (Illumina, San Diego, CA, USA) to generate 2 × 300 bp paired-end reads. 15% PhiX fragment library was added to increase sample diversity.

### Quality control and filtering

PCR primer sequences and Illumina adapter sequences were trimmed using Cutadapt (version 1.2.1), sequencing errors were corrected using the SPAdes sequence assembler (version 3.1.0), and sequences outside the 200-750 bp range were removed. Final sequences were analysed using a custom pipeline based on QIIME 1.9.0. Amplicon sequences in each sample were assigned to clusters, based on 97% similarity threshold, using the Silva database (release 123) and the VSEARCH 1.1.3 and SWARM clustering algorithms, merging the results. Potential chimeric sequences were discarded. In total, 48,991,273 analysed sequences were clustered in 75,643 different operational taxonomic units (OTUs).

### Analysis of taxonomic composition

Taxonomic assignment was carried out using QIIME and the RDP classifier. OTUs were removed from the dataset if they appeared in fewer than ten samples. Samples were rarefied to 135,000 sequences per sample leading to exclusion of 17 samples; consequently, 242 samples remained in the final dataset.

### Comparison of microbial diversity between different foot-health groups

Alpha diversity was assessed using the Shannon and Simpson diversity indices for species evenness and the Chao 1 index for species richness. Data were analysed using both the t-test and the Wilcoxon rank sum test to compare foot health groups to each other.

Beta diversity was assessed at the OTU level with unweighted and weighted UniFrac phylogenetic distances using QIIME (v2). Phylogenetic distance matrices were analysed using Principal Coordinates Analysis (PCoA) and plots were generated and visualized in EMPeror.

Beta diversity was further investigated using pairwise Permutational Multivariate Analysis of Variance (PERMANOVA) with 999 permutations at farm level after restricting the dataset to the HtHt and HtIn groups of primary interest. Differences in beta diversity between farms in the HtHt and HtIn foot health groups was also further investigated using DEICODE in QIIME (v2) to carry out Robust Aitchison Principal Coordinates Analysis [16]. Qualitative data generated was used to construct a biplot showing which OTUs were most influencing beta diversity [17].

### Comparing the foot-skin microbiome between HtHt, HtIn and InIn foot-health groups

For genus-level comparison between samples from cows with different BDD status, the dataset was restricted to the twenty most prevalent phyla and genera with a minimum 0.5% mean relative abundance. Log fold changes (Log10) were calculated for each sample and mean relative abundances were logit transformed. Response screening was carried out in JMP Pro 12 (SAS Institute Inc., Cary, NC) to evaluate the differences in OTU (genus level assignments) relative abundance between the samples with different foot-health status. P-values were adjusted for False discovery rate (FDR) and presented as Robust FDR LogWorth. Log fold change of genera was plotted versus Robust FDR LogWorth using Y mean relative abundance as circle size, and effect size as colouring.

Because of the limitations of response screening as a method for analysing compositional data [18], data were also analysed using the Songbird package for multinomial regression in QIIME 2 to rank differential abundance of OTUs in the foot-health groups of primary interest (HtHt and HtIn) for comparison. Importantly, this comparison was made while controlling for the potentially confounding factors of farm and season.

### Co-occurrence analysis

To identify the ecological interactions [19] among the microbial taxa in the samples, co-occurrence analysis for the previously identified OTUs was performed using the SParse InversE Covariance Estimation for Ecological ASsociation Inference tool (SPIECEASI) [20]. OTUs below 0.005% of the total frequency were excluded (as proposed by Bokulich et al., 2013 [21]). This approach reduced the number of OTUs from 75,643 to 3,039. Network analysis was carried out using Cytoscape Version 3.6.1 (USA). Comparisons were made between HtHt and HtIn groups to identify mutualistic or competitive interactions that differ between the two disease groups at phylum level (filtered to include the six phyla that contribute > 1% of total OTUs), and therefore may influence development of BDD lesions. Network statistics were computed using NetworkAnalyzer in Cytoscape. Genus level analysis was restricted to the following genera and their adjacent nodes (this was guided by results obtained from response screening analysis): *Succiniclasticum* spp., *Porphyromonas* spp., *Acholeplasma* spp., *Anaerococcus* spp., *Fastidiosipila* spp., *Prevotella* spp. and *Peptoclostridium* spp., which were found to be more prevalent in HtIn samples compared to HtHt samples, and *Brachybacterium* spp. and *Macrococcus* spp. which were found to be more prevalent in HtHt samples. *Treponema* spp, were also investigated as they were absent from the top 20 most prevalent genera in HtHt samples, but present in the top 20 for HtIn samples, and they are widely considered to be one of the key pathogens in BDD pathogenesis [1].

### Shotgun metagenomic analysis

To maximise the chances of achieving sufficient sequencing depth, cows were selected at random from those whose previous 16SrRNA samples had a DNA content of > 5 ng/μl after the initial DNA extraction, as measured using the Qubit™ dsDNA HS Assay Kit. Microbial DNA was extracted from a second set of swabs that had been collected parallel to those used in the marker gene analysis. Dna extraction negative controls were not included in this process. The DNA extraction method was the same, using the PureLink™ Microbiome DNA Kit (Invitrogen, Carlsbad, CA, USA) according to the manufacturer’s instructions. Agarose gel electrophoresis was carried out using SYBR green as the nucleic acid stain (Thermo Fisher Scientific Fair Lawn, NJ, USA) to ensure presence of clear DNA bands. Library preparation was carried out on gDNA samples using the Nextera XT kit (Illumina). gDNA input was quantified using Qubit™ to ensure 1 ng of each sample was submitted for tagmentation. The libraries were sequenced on an Illumina HiSeq 4000 platform using sequencing by synthesis (SBS) technology to generate 2 × 150 bp paired-end reads.

### Quality control and filtering of shotgun metagenomic sequences

Data files were demultiplexed and converted to FASTQ format using Casava v.1.8.2 (Illumina). FASTQ files were trimmed using option _O3 Cutadapt version 1.2.1 [22] to exclude those matching Illumina adaptor sequences by $\ge$ 3 bp at the 3’ end. Reads were further trimmed to remove low quality bases, using Sickle version 1.200 with a minimum window quality score of 20. After trimming, reads shorter than 20 bp were removed, and single reads were excluded as length distributions showed they were of poor quality. Host reads were removed following alignment against the host *Bos taurus* genome using Bowtie2 v2.2.6 [23]: read pairs where one or both reads aligned were removed. The remaining reads in pairs were merged using PEAR v0.9.11 [24] to form a single long read based on overlapping homology. Those that could not be merged in this way were concatenated with an intervening N-base. Resulting sequences underwent taxonomic assignment using Kraken v0.10.6 [25] and results were filtered using a confidence threshold of 0.1. Results were analysed using Linear discriminant analysis effect size (LefSe) [26] to determine taxa most likely to explain differences between the two classes HtHt and HtIn. The HUMAnN2 search strategy [27] was used to functionally annotate read data and abstracts to show biological pathway abundance and completeness. Finally, reads that did not align to their pangenomes using this strategy were submitted to a protein database (UniRef) for translated searching [28]. The gene families identified were further analysed using the MetaCyc database to reconstruct and quantify complete metabolic pathways [29].

### Genome Wide Association and regional heritability mapping study of foot skin microbiota related traits

Animal sampling and genotyping are described by Sánchez-Molano et al. [14]. Phenotypic traits (*n* = 10) analysed here included three different alpha diversity indices; Chao1, Shannon, Simpson indices, and relative abundances of seven genera; *Porphyromonas* spp., Clostridiales Family XI, *Fastidiosipila* spp., *Peptoclostridium* spp., *Macrococcus* spp., *Treponema* spp., and genera of the phylum Bacteroidetes.

The Genomic relationship matrix (GRM) was computed using GEMMA [29] and principal component analysis (PCA) was used to find out any genetic structure of the cow population. This population structure was accounted for in GWA models by automatically fitting the GRM as part of the polygenic effect, whereas in RHM analysis the first 7 PCs were fitted to account for this structure (RHM analyses failed to converge when the GRM was fitted); further correction for the inflation factor (*λ*) was applied as described by Amin et al. [30]. REACTA [31] was first used to assess the full genomic variance for each trait with a general explanatory analysis. GWA and RHM was performed using GEMMA [29].

## Results

A summary of the number of samples included in each foot-health group, and the farm of origin, can be found in Supplementary Information Table 1.

**Table 1:** Alpha diversity metrics showing species richness and evenness. Statistically significant *P*-values are shown in bold (*P* < 0.05), including the differences with *P* < 0.1. (HtHt: The cows which remained healthy throughout the study, HtIn: The cows which were healthy at initial sampling, then developed BDD, InIn: The cows which had BDD at all inspection points, InHt: The cows which had BDD at initial sampling then recovered, BL_HtHt: The cows which had healthy BL feet during the study, BL_HtIn: The cows which were healthy at sampling, then developed BDD on their BL feet, BL_InIn: The cows which had BDD in their BL feet at all sampling points, BL_InHt: The cows which had BDD in their BL feet at initial sampling, then recovered. *P*-value: *P* value of t-test, SE: standard error, N_*P*_value: *P*-value of nonparametric Wilcoxon rank sum tests)

	n	Chao1	SE	*P*-value	N_*P*-value	Shannon	SE	*P*-value	N_*P*-value	Simpson	SE	*P*-value	N_*P*-value
HtHt	112	13412.22	181.74	0.5575	0.8406	10.61	0.06	**0.0903**	0.8319	0.05	0.002	0.1899	0.2888
HtIn	48	13217.19	277.61			10.41	0.1			0.04	0.003		
HtHt	112	13412.22	218.41	**0.0165**	**0.0839**	10.61	0.08	**0.0002**	**0.0048**	0.05	0.002	**0.0494**	**0.0595**
InIn	59	12511.4	300.93			10.12	0.11			0.04	0.003		
HtHt	112	13412.2	190.76	0.6445	0.7079	10.61	0.06	**0.0166**	0.21	0.05	0.002	**0.0647**	**0.0452**
InHt	16	13162.6	504.69			10.2	0.16			0.04	0.005		
InIn	59	12511.4	404.08	0.459	0.3257	10.12	0.15	0.8114	0.7661	0.04	0.003	0.5691	0.4532
InHt	16	13162.6	775.94			10.2	0.29			0.04	0.005		
BL_HtHt	148	13532.40	173.38	**0.0167**	0.1053	10.60	0.06	**0.0116**	0.2616	0.05	0.002	0.5797	0.6584
BL_HtIn	41	12633.60	329.41			10.27	0.11			0.04	0.003		
BL_HtHt	148	13532.40	179.65	**0.0052**	**0.0369**	10.60	0.06	**0.0001**	**0.0002**	0.05	0.002	**0.0107**	**0.0152**
BL_InIn	37	12396.40	359.29			10.07	0.12			0.04	0.003		
BL_HtHt	148	13532.40	166.97	**0.0125**	0.1450	10.60	0.06	**0.0001**	**0.0056**	0.05	0.002	**0.0014**	**0.0014**
BL_InHt	9	11769.90	677.08			9.52	0.22			0.02	0.007		
BL_InIn	37	12396.40	506.10	0.5868	0.8139	10.07	0.19	0.2159	0.2505	0.04	0.003	0.0513	0.0509
BL_InHt	9	11769.90	1026.20			9.52	0.39			0.02	0.006		

### Taxonomic composition

Taxonomic composition was examined for HtHt and HtIn groups at phylum and genus level. Dominant phyla in descending order of abundance were Firmicutes, Bacteroidetes, Proteobacteria and Actinobacteria. For HtIn samples, Tenericutes and Spirochaetae also accounted for > 1% of bacteria identified. The most abundant family identified were Ruminococcaceae from the phyla Firmicutes. This was reflected at genus level, where the most abundant identified genera were *Ruminococcaceae* UCG-005 and UCG-010. The potential pathogens *Porphyromonas* spp. and *Treponema* spp. were identified at higher relative abundance in the HtIn group compared to the HtHt group (Supplementary Information Fig. 1).

### Comparison of microbial diversity between foot-health groups

Differences in sample richness and evenness were identified between foot-health groups. Alpha-diversity metrics overall suggested that HtHt samples had significantly greater microbial diversity than InIn or InHt samples, and a tendency to greater microbial diversity than HtIn samples (Table 1).

**Fig. 2:**
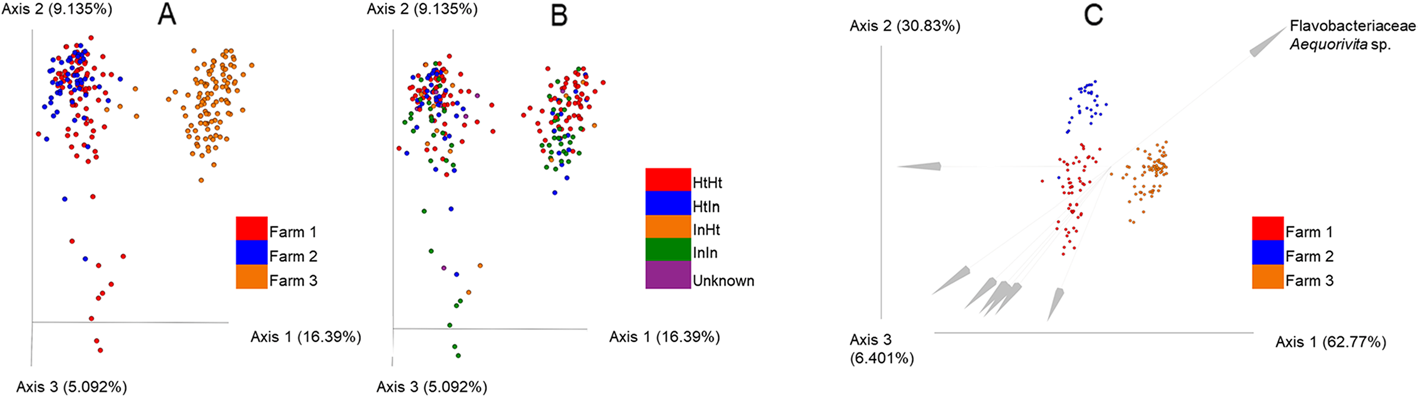
Unweighted unifrac distances showing beta diversity (A) by farm, and (B) by foot health group, with (C) the results from DEICODE analysis identifying the taxon most responsible for Farm 3 clustering away from Farms 1 and 2

Graphs showing PCoA of unweighted UniFrac distances displayed by farm and by foot-health group are shown in Figs. 2A and 2B. Farm three clusters away from farms one and two, suggesting the phylogeny of farm three samples differs from the other farms. Graphs showing PCoA of weighted UniFrac distances for farms and foot-health groups are available in Supplementary Information Fig. 2.

Because of these differences in beta-diversity found at farm level, PERMANOVA was used to test for significant differences in beta-diversity between HtHt and HtIn foot-health groups for each farm separately. Results show differences in farm one for both unweighted and weighted UniFrac distances (pseudo F = 1.528, *P* = 0.014, r^2^ = 0.03) and pseudo F = 4.409, *P* = 0.01, r^2^ = 0.08 respectively) and in farm three for unweighted UniFrac distances (pseudo F = 1.859, *P* = 0.002, r^2^ = 0.03). No significant differences were found for farm two, or for weighted UniFrac distance for farm three.

The biplot resulting from DEICODE analysis identified OTU32700 as most influential in causing farm three samples to cluster away from Farms 1 and 2 (Fig. 2C). This OTU was identified as coming from the Flavobacteriaceae family: specifically, *Aequorivita* spp.

### Comparing the composition of the foot-skin microbiome of the HtHt foot-health group to the HtIn and InIn groups

**Fig. 3:**
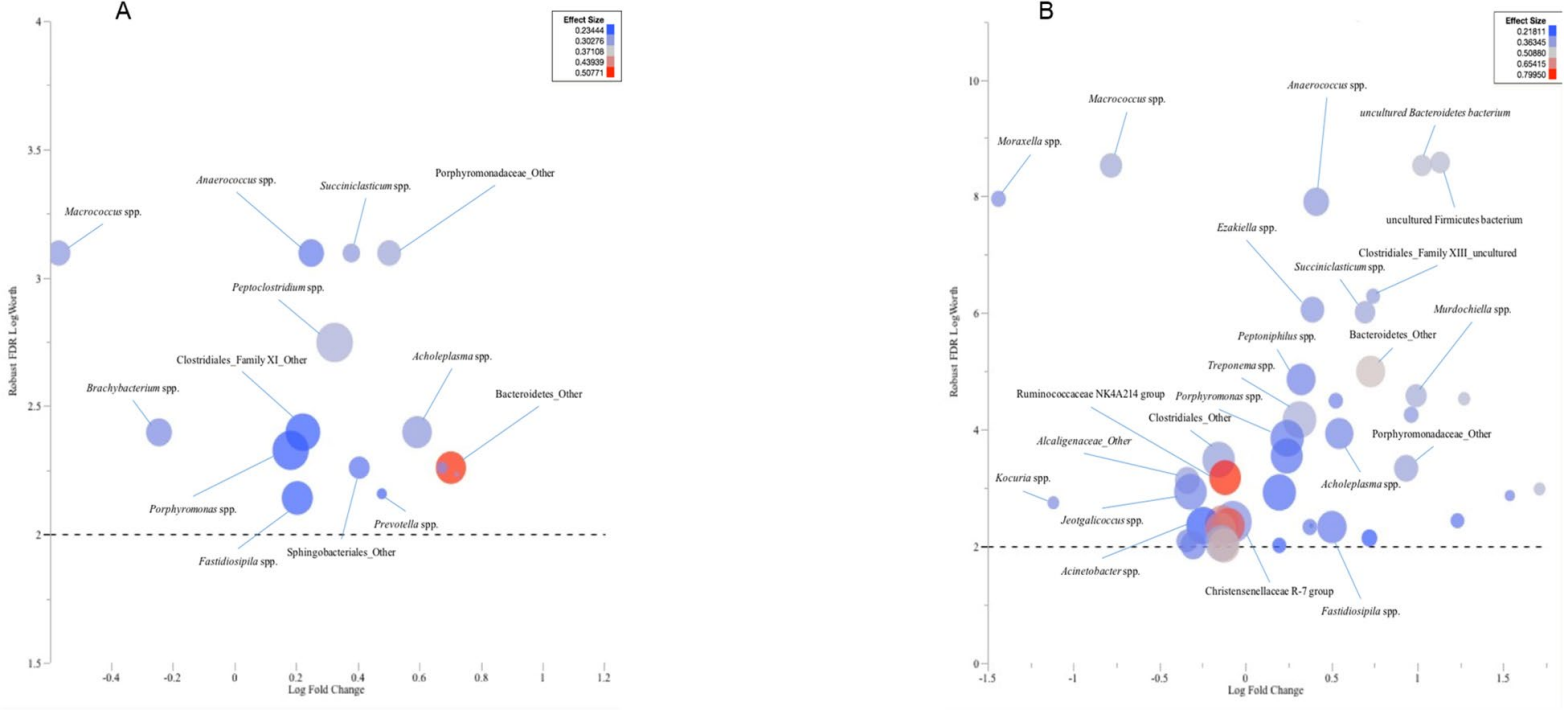
Results of response screening comparing the microbiota profile at genus-level of HtIn samples (3A) and InIn samples (3B) relative to HtHt samples versus corrected robust false discovery rate (FDR) logWorth (i.e. log10P). The dashed line shows the P-values (0.01) adjusted for FDR. The size of circles represents mean relative abundance of each genus, and colour represents the effect size

Response screening showed that compared to HtHt samples, the HtIn samples showed higher prevalence of the genera *Succiniclasticum* spp., *Porphyromonas* spp., *Acholeplasma* spp., *Anaerococcus* spp., *Fastidiosipila* spp., *Peptoclostridium* spp. and *Prevotella* spp. HtIn samples showed lower prevalence of the genera *Macrococcus* spp. and *Brachybacterium* spp (Fig. 3A). InIn samples showed higher prevalence of the genera *Succiniclasticum* spp., *Porphyromonas* spp., *Treponema* spp., *Acholeplasma* spp., *Anaerococcus* spp., *Fastidiosipila* spp., *Peptoclostridium* spp., *Murdochiella* spp., *Ezakiella* spp. and *Peptoniphilus* spp. compared to HtHt samples, and lower prevalence of *Macrococcus* spp., *Moraxella* spp., *Kocuria* spp., *Jeotgalicoccus* spp., *Acinetobacter* spp., and the Ruminococcaceae NK4A214 group (Fig. 3B).

Songbird analysis identified the same genera that were found to be more abundant in HtIn samples compared to the HtHt samples in response screening to be highly associated with the HtIn group (except for *Anaerococcus* spp.). *Treponema* spp. were the fourth most highly associated taxa with HtIn samples; however, *Macrococcus* spp. and *Brachybacterium* spp. were not found in the top twenty-five taxa most associated with HtHt samples in this analysis. Further taxa strongly associated with either HtIn or HtHt groups are shown in Table 2.

**Table 2:** Twenty-five taxa most associated with the HtHt and HtIn foot health groups, as determined using Songbird analysis

	HtHt	HtIn
1	Nosocomiicoccus	Acholeplasma
2	Flavobacterium	Mycoplasma
3	Oceanobacter	Family XIII AD3011 group
4	Ruminococcaceae UCG-010	Treponema 2
5	Nocardiodes	Murdochiella
6	Perlucidibaca	Fretibacterium
7	Salinococcus	uncultured Firmicutes bacterium
8	Bacteroides	Succiniclasticum
9	Prevotellaceae UCG-004	Fastidiosipila
10	Ruminococcaceae UCG-014	Catonella
11	Ruminococcaceae UCG-013	Peptoclostridium
12	Lachnoclostridium	Prevotella
13	Erysipelotrichaceae	Campylobacter
14	Christensenellaceae	Roseburia
15	Oceanobacillus	Peptostreptococcus
16	Clostridium sensu stricto	Ruminococcaceae UCG-014
17	Phocaeicola	Uncultured Bacteroidetes bacterium
18	Corynebacterium 1	Arcanobacterium
19	Chryseobacterium	Parvimonas
20	Ruminococcus 2	Lachnospiraceae AC2044 group
21	Epulopiscium	Prevotella
22	Psychrobacter	Porphyromonas
23	Ruminiclostridium	Cellvibrio
24	Marmoricola	Corynebacterium 1
25	Nocardioides	Fusibacter

### Co-occurrence analysis

Both HtHt and HtIn sample groups (filtered to the six phyla contributing > 1% of nodes) had low network density and network centralisation with no hub nodes identified. Network heterogeneity was slightly lower for HtIn groups with a higher average number of neighbours. HtIn groups had fewer connected components, shorter characteristic path length and smaller network diameter despite larger number of nodes, showing stronger connectivity and shorter expected distances between nodes (Supplementary Information Table 2 and Supplementary Information Fig. 3A and B).

When the genera that were known to differ in relative abundance or overall presence between groups were selected and examined alongside their adjacent nodes, it was noted that more negative interactions existed in HtIn groups. There was no clear pattern to the identity of adjacent nodes, but it was apparent that the negative interactions originated from the eight genera that were more abundant in the HtIn samples compared to the HtHt samples (Supplementary Information 3C and D).

### Shotgun metagenomic analysis

**Fig. 4:**
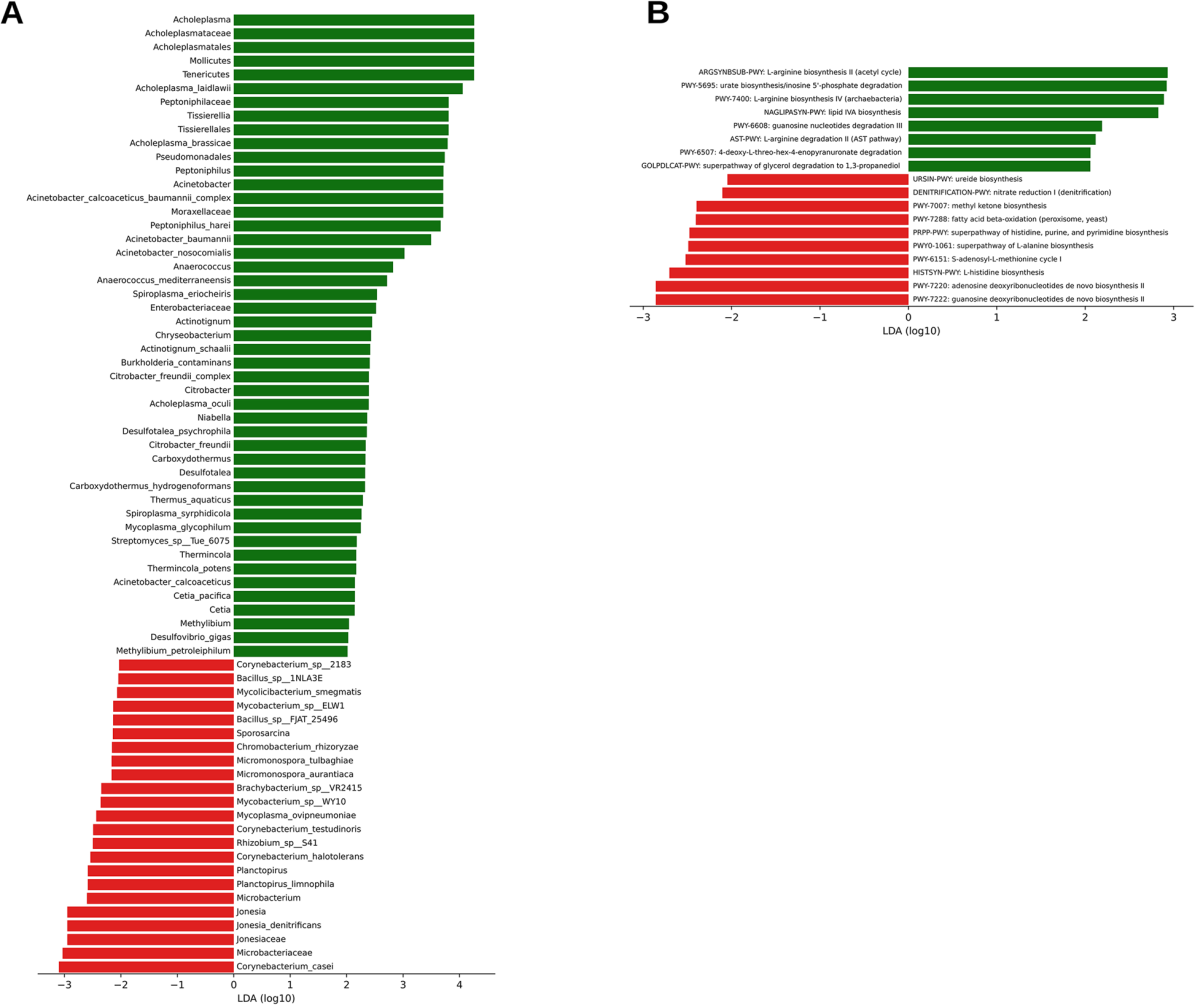
4A. LefSe analysis showing taxa that were found to be significantly different between the HtHt (0, in red) group and HtIn (1, in green) group. 4B. Biologically relevant differences between HtHt (0, in red) and HtIn (1, in green) samples in functional pathways identified using HUMAnN2 and LEfSe analysis

Taxonomic assignment of reads was low and therefore the significance of these findings is uncertain; however, there may be some agreement with the 16S rRNA sequencing analysis in finding increased *Acholeplasma* spp. in HtIn samples and increased *Brachybacterium sp.* in HtHt samples (Fig. 4A).

Ten functional pathways were identified as significantly more abundant in the HtHt group (Fig. 4B). All were metabolic pathways for synthesis or degradation of amino acids or fatty acids, or pathways involved in nucleotide synthesis. Eight functional pathways were identified as significantly more abundant in the HtIn group. Three of these pathways were associated with degradation of nucleotides and one indicated production of 4-deoxy-L-*threo*-hex-4enopyranuronate, which is a uronic acid resulting from the degradation of many polymers. These include plant polymers such as pectin and gellan, but also important components of connective tissue such as heparin, heparin sulfate, hyaluronan and chondroitin sulfate [32] (Fig. 4B). Despite detection of some differences in individual functional pathways, no overall differences in abundance for gene families in the GO slim categories of biological processes, cellular components, or molecular functions were detected (Supplementary Information Fig. 4A, B and C respectively).

### Genome-Wide Association study and regional heritability mapping of foot skin microbiota related traits

Table 3 shows the total genomic variance and heritability estimates for the relative abundance of *Peptoclostridium* spp. and *Treponema* spp. All other examined traits are not included due to total genomic variance estimates being non-significantly different from zero. The heritabilities for the relative abundances of *Peptoclostridium* spp. and *Treponema* spp. were 0.59 ± 0.18 and 0.52 ± 0.00, respectively.

**Table 3:** Significant (*P* < 0.05) estimates of heritability and variance. Genomic heritabilities (*h*^*2*^), genomic variance (*Vg*) estimated together with their standard errors and number of records (*N*)

Trait	Vg	h^2^	*P value*	N
Mean Relative abundance of *Peptoclostridium* spp.	0.000053 ± 0.000019	0.59 ± 0.18	7E-04	236
Mean Relative abundance of *Treponema* spp.	0.000129 ± 0.000055	0.52 ± 0.00	0.007	236

**Fig. 5:**
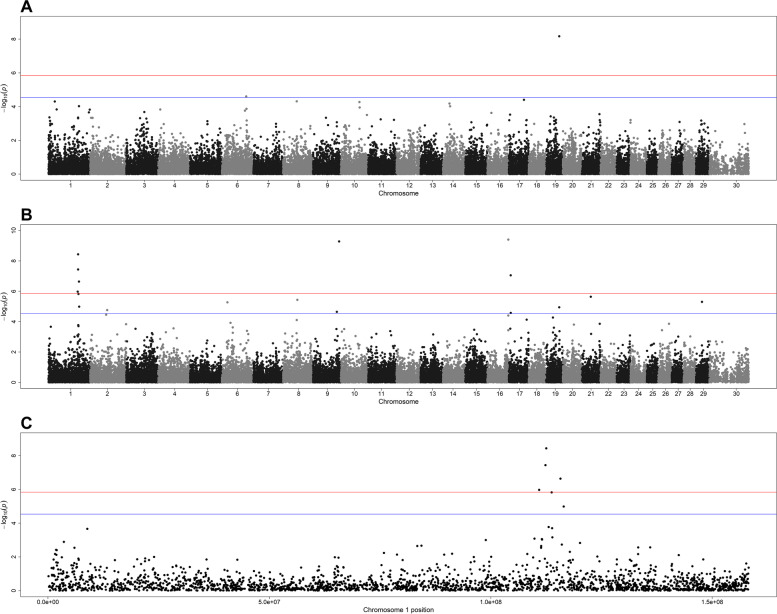
Manhattan plots from genome-wide association analysis of relative abundance of *Peptoclostridium* spp. (A) and relative abundance of *Treponema* spp. (B). Figure C shows a closer look at the association analysis of SNPs in *Bos taurus* autosome 1 (BTA1) with relative abundance of *Treponema* spp. The red line represents the genome-wide significance (Bonferroni corrected, so 0.05 divided by the number of SNPs) and the blue line represents the suggestive threshold (Bonferroni corrected, so 0.1 divided by the number of SNPs). In these plots, genomic coordinates are shown along the x-axis (ordered by chromosome and position in base pairs), and the negative logarithm of the association p-value for each SNP is shown on the y-axis. X-axis labels in A and B correspond to the chromosome number while in C correspond to SNP position (in base pairs) within chromosome 1

Suggestive and significant SNPs associated with these two traits after GWA analyses are shown in Table 4. The association between individual SNPs and relative abundances of *Peptoclostridium* spp. and *Treponema* spp. are also shown in Manhattan plots (Fig. 5).

**Table 4:** Summary of genome-wide suggestive and significant SNPs for the traits Mean Relative Abundance of *Peptoclostridium* and *Treponema* spp., including their positions on corresponding chromosomes (BTAs) and significance level (*P*-value)

Trait	BTA	Position (BP)	*P*-value	Significance
Mean Relative abundance of *Peptoclostridium* spp.	6	92217233	2.49E-05	Suggestive
	19	50478941	6.78E-09	Significant
Mean Relative abundance of *Treponema* spp.	1	112526671	3.65E-09	Significant
	1	112344219	3.65E-08	Significant
	1	115738119	2.28E-07	Significant
	1	110924093	1.06E-06	Significant
	1	113745976	1.49E-06	Suggestive
	1	116472073	1.03E-05	Suggestive
	2	64462072	1.74E-05	Suggestive
	6	20730690	5.32E-06	Suggestive
	8	54239367	3.67E-06	Suggestive
	9	99334002	5.22E-10	Significant
	9	90719582	2.26E-05	Suggestive
	16	79449472	3.93E-10	Significant
	17	7185597	8.88E-08	Significant
	17	7399427	2.65E-05	Suggestive
	19	50478941	1.14E-05	Suggestive
	21	34339514	2.28E-06	Suggestive
	29	23162838	4.97E-06	Suggestive

**Fig. 6:**
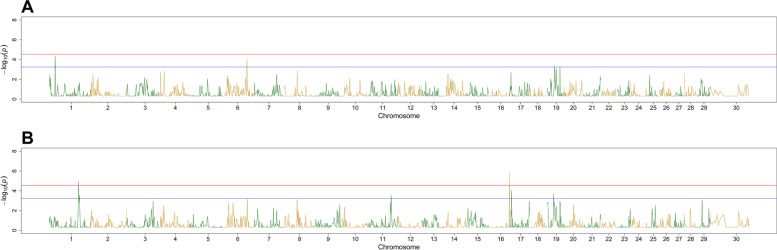
Manhattan plots from regional heritability mapping results for relative abundance of *Peptoclostridium* spp. (A) and relative abundance of *Treponema* spp. (B) Red line represents the genome-wide significance threshold (Bonferroni corrected, so 0.05 divided by the number of SNPs) and blue line represents the suggestive threshold (Bonferroni corrected, so 0.1 divided by the number of SNPs). In these plots, genomic coordinates are shown along the x-axis (ordered by chromosome and position in base pairs), and the negative logarithm of the association *p*-value for each region is shown on the y-axis

The RHM analysis identified one suggestive region on both BTA1 and BTA6 and two suggestive regions on BTA19 for the trait relative abundance of *Peptoclostridium* spp. For the trait relative abundance of *Treponema* spp. RHM results indicated one region on both BTA1 and BTA16 with genome-wide significance (Fig. 6A), besides suggestive regions on BTA1, BTA11, BTA17 and BTA19 (Fig. 6B).

The results of GWA and RHM analyses were combined, and a consensus table of genomic regions was created with the start and ending positions of each region on corresponding BTAs, the proportion of genomic variance explained by each region, and potential candidate genes neighbouring the regions (Table 5). Interestingly, some of the identified regions explained a substantial proportion of the genomic variance with the region in BTA19 explaining 28.07% of the genomic variance for the relative abundance of *Peptoclostridium* spp. and the region in BTA16 explaining 34.78% of the genomic variance for the relative abundance of *Treponema* spp.

**Table 5:** Summary of the consensus genomic regions identified in the GWA and RHM analyses including the proportion of genomic variance (*V*_*g*_) explained by the detected region, and candidate genes in these regions

Traits	BTA	Start Position (BP)	Ending position (BP)	*P-*value	Proportion of Vg explained	Candidate Genes
Mean Relative abundance of *Peptoclostridium* spp.	19	5E + 07	5.1E + 07	0.00057	28.07%	*ZNF750*
Mean Relative abundance of *Treponema* spp.	1	1.1E + 08	1.1E + 08	1.17E-05	9.88%	*GMPS* and *PLCH1*
	1	1.2E + 08	1.2E + 08	0.00028	1.97%	*MBNL1*
	16	7.9E + 07	8E + 07	1.23E-06	34.78%	*PTPRC*
	17	7185597	8690167	9.14E-05	7.11%	*LRBA*

## Discussion

Our prospective cohort study allowed us to show differences in the taxonomy, function and ecological interactions of commensal microbiota of the foot skin between dairy cows that remained healthy and those that went on to develop BDD in the future. This is the first study to investigate associations between the bovine foot skin microbiome (described by both 16S rRNA amplicon and shotgun metagenomics sequencing) with future development of BDD lesions as until now all studies associating bovine foot skin microbiomes with BDD have been cross-sectional. To achieve this, we employed swabbing of the foot skin as opposed to foot skin biopsies which would have disrupted the skin integrity, “artificially” increasing the chances of future BDD infection. Swabbing of the bovine foot skin allowed us to study natural disease progression in a “real life” setting (commercial dairy farms). Additionally, we identify for the first time associations between the bovine host genetics and the relative abundance of bacterial genera important in the development of BDD lesions.

Previous studies have found Firmicutes and Actinobacteria to be dominant in healthy skin, whereas we identified Bacteroidetes and Proteobacteria to be more abundant than Actinobacteria [1, 3, 6]. Treponema spp. were present at low levels even in samples from healthy cows which is consistent with previous studies that have shown their abundance increasing in active BDD lesions [1, 6, 33]. *Ruminococcaceae* from the Firmicutes phylum were the most abundant family and are also dominant in the bovine faecal microbiome [34]. We interpret that there is likely to be considerable overlap between foot-skin and faecal microbiota in housed dairy cattle as part of an environmental effect.

Although HtHt samples had greater species richness and evenness, statistically significant differences in alpha diversity were observed only when comparing samples from feet that were healthy and those that were infected at the time of sampling. Reduced alpha diversity can be the result of loss of beneficial microbes or overgrowth of harmful microbes, or a general loss of microbial diversity. It has been suggested that a combination of all three of these changes may be required to promote disease [35]. This phenomenon has been observed in other polybacterial diseases, for example low bacterial diversity has been associated with skin inflammation in ovine footrot [36] and has also been observed in bovine mastitis cases [37]. The use of probiotics to modify the gut microbiota has become an accepted concept for improving intestinal health in people [38]. Although similar research pertaining to the skin microbiome is in the early stages, there is evidence that topical application of Lactobacillus bacteria and ammonia-oxidizing and nitrifying bacteria may help to maintain a healthy skin microbiome [39]. There is also some indication that probiotics may be useful for treatment of atopic dermatitis in children, and there may be mechanisms by which using probiotics to influence the gut microbiome may exert beneficial effects on the skin [40]. It is possible that a similar concept for preventative treatment targeting the maintenance of microbial diversity in the bovine foot-skin may be successful in halting the development of BDD lesions and is a promising area for future research. This could potentially be achieved using pro- or pre- biotic footbathing; a management practice already routinely carried out on cattle farms using biocides for BDD prevention.

Differences in unweighted UniFrac distances were noted between farm three and the other two farms suggesting that the foot-skin habitat of cows on farm three differs from those on the other two farms. These differences in the foot-skin microenvironment might be caused by differences in management systems which could not be controlled in this study. In our case the main differences in farm management between farm 3 and farms 1 and 2 that could explain the observed differences in foot skin microbiota profiles were the following: sampled animals in farm 3 were housed in deep sand bedded cubicles and were walking through a 2% formalin footbath three times a week; sampled animals in farms 1 and 2 were housed in a deep straw bedded yard and were not walking through a footbath when they were not milked.

PERMANOVA analysis of unweighted UniFrac distances on farm three also showed that beta diversity of HtHt samples differed from HtIn samples, suggesting that there may be differences in the skin micro-environment between the foot-health groups driven by different founding populations of microbes. Farm one samples also showed this pattern as well as a statistically significant difference in weighted UniFrac distances, showing quantitative differences in relative taxon abundance between HtHt and HtIn groups. Differences between HtHt and HtIn animals could also be associated with compromised skin integrity which has been shown to be essential for the development of BDD in experimental challenge models [41]. We did not detect skin damage in HtHt or HtIn animals at the time of sampling but that doesn’t preclude the presence of skin damage that was still macroscopically undetectable. The Songbird analysis identified *Mycoplasma* spp., *Acholeplasma* spp. and *Treponema* spp. in association with the HtIn foot health group (Table 2). *Mycoplasma* spp. have been previously identified as associated with bovine digital dermatitis [1, 42], as have *Acholeplasma* spp. [1]. Songbird analysis also identified *Murdochiella* spp., which have been previously associated with wounds and bacterial vaginosis in people, but are previously unreported for BDD [43, 44].

Shotgun metagenomic analysis showed differences in taxa present in HtHt compared to HtIn samples. Functional differences in the microbiome, for example increases in genes for flagellar motility and zinc and copper resistance, have been previously reported in biopsies taken from BDD lesions compared to healthy skin. Our data may suggest an increase in pathways relating to degradation of connective tissues in HtIn samples; perhaps early evidence of upregulation of pathogenic genes that could initiate skin damage. Overall significant differences in the abundance of gene families responsible were not detected. Either functional differences in the skin microbiome do not materialise before development of morphological lesions or are undetectable from our data, perhaps because samples came only from the skin surface, or possibly due to small sample sizes and a large percentage of unassigned sequences.

We show for the first time here that certain regions in the bovine genome may harbour genes associated with the relative abundance of members of the foot skin microbial communities. The proportion of the total genomic variance explained by the detected regions for each trait ranged from 1.97% to 34.78% suggesting a partially oligogenic architecture; however this could be overestimated due to the Beavis effect [45]. The region associated with relative abundance of *Treponema* spp. on BTA1 explains 9.88% of the total genomic variance and includes the genes *GMPS* and *PLCH1*. *GMPS* encodes guanine monophosphate synthetase which plays a role in de novo synthesis of guanine nucleotides; the cyclic form of GMP was shown to be associated with immune signalling pathways [46, 47]. *PLCH1* is a member of the phospholipase enzyme family that generates the secondary messengers inositol 1,4,5-trisphosphate (IP3) and diacylglycerol (DAG) by cleaving phosphatidylinositol 4,5-bisphosphate (PtdIns(4,5)P2). Phospholipases were shown to be involved in inflammation mechanisms [48], especially the expression of *PLCH1* which was shown to be downregulated by lipopolysaccharides (LPS) [49] which are found in the outer membrane of Gram-negative bacteria [50]. The region associated with relative abundance of *Treponema* spp. on BTA16 explains 34.78% of the total genomic variance and includes the gene *PTPRC* encoding a transmembrane tyrosine phosphatase which was shown to be upregulated after administration of external bacteria to the intestine of mice [51, 52]. On BTA17, the region associated with the relative abundance of *Treponema* spp. explains 7.11% of the total genomic variance and harbours the LPS-responsive beige-like anchor gene (*LRBA*) which is expressed in immune cells after stimulation by LPS [53]. Mutations on the *LRBA* gene were shown to be associated with immune system related disorders such as immunodeficiency, inflammatory bowel disease [54], and autoimmunity [55]. On BTA19, the region associated with relative abundance of *Peptoclostridium* spp. explains 28.07% of the total genomic variance and harbours the gene *ZNF750* which encodes a putative C2H2 zinc finger protein which was shown to be associated with the skin disorders Seborrhoea-like dermatitis [56] and familial psoriasis [57]. In addition, increased dietary zinc was shown to be associated with reduced BDD incidence in dairy cows [58]. Admittedly, the above proportions of genomic variance accounted for by genomic regions may be somewhat inflated and more research is needed to refine estimates of the collective impact of the identified regions. Nevertheless, our results provide evidence of host genetic control of two genera in the foot skin microbiota profile, which, combined with the association of the latter with BDD lesion development offer new insights into a complex relationship that can be exploited in selective breeding programmes aiming to enhance bovine foot health.

## Conclusion

We have shown for the first time that certain members of the bovine foot-skin microbiota are associated with host genotype and the future development of BDD lesions. 16S rRNA gene sequencing analysis of swabs taken from morphologically normal foot-skin surfaces identified taxa associated with future development of BDD lesions and taxa which appeared protective. Shotgun metagenomic analysis corroborated *Acholeplasma* spp. as detrimental, and *Brachybacterium* spp. as protective, and identified higher abundance of genes that could be associated with collagen degradation in samples from cows that subsequently developed BDD lesions. Finally, we identified regions of the bovine genome associated with relative abundance of *Treponema* spp. and *Peptoclostridium* spp., two of the genera identified by 16S rRNA sequencing as associated with future development of BDD lesions. Collectively this work shows the relevance of the bovine foot-skin microbiota to the development of BDD.

## Supplementary Information

**Additional file 1.**

## Data Availability

Sequences are available on the MG-RAST metagenomics analysis server athttps://www.mg-rast.org/linkin.cgi?project=mgp91792. The 16S rRNA gene amplicon sequences have been deposited at the NCBI BioProject database (BioProject ID PRJNA702425). The genotype data has been uploaded to a public repository hosted by the University of Edinburgh. Genotypes are therefore publicly available and can be obtained from: Edinburgh DataShare (University of Edinburgh), https://datashare.is.ed. ac.uk/handle/10283/3409.
