# Supplementary material for: The bovine foot skin microbiota is associated with host genotype and the development of infectious digital dermatitis lesions
Source: Microbiome. 2023 Jan 10;11:4. doi: 10.1186/s40168-022-01440-7 (PMC9830885; doi:10.1186/s40168-022-01440-7)

SUPPLEMENTARY INFORMATION

Detailed methods

DNA extraction, 16S rRNA gene amplification, and sequencing

Microbial DNA was extracted from collected swabs using the PureLink™ Microbiome DNA Kit (Invitrogen, Carlsbad, CA, USA) which utilizes chemical, heat and bead-beating cell lysis prior to purification. Extracted DNA samples were stored at -20°C until amplification for sequencing. DNA was also extracted from two swabs that were not used to sample cows; these served as negative controls. Amplification of the V3-V4 hypervariable region of the 16S rRNA gene for sequencing was conducted using Illumina_16S_341F and Illumina_16S_805R universal primers with adapter sequences.The first step PCR mastermix contained 1.25µl of amplicon PCR forward and reverse primers (2.5µM) and 12.5µl of NEBNext High-Fidelity 2X PCR Master Mix (New England Biolabs, Ipswich, MA, USA). Reaction conditions were 95°C initial denaturation for 3 min, followed by 12 cycles of 95°C for 30 s, 62.3°C for 30 s, and 72°C for 30 s, and a final extension at 72°C for 5 min. Negative (Nuclease-Free Water (not DEPC-Treated) Thermo) and positive controls (ZymoBIOMICS™ Microbial Community DNA Standard) were also amplified. PCR products were cleaned with Agencourt AMPure XP beads (Beckman Coulter Genomics, Fullerton, CA, USA) following the manufacturer's protocol. In a second PCR step, dual indexes and Illumina sequencing adapters were attached using 7.5µl of amplicon PCR product DNA, 2.5µl of Illumina Nextera XT Index Primer 1 (N7xx), 2.5µl

of Nextera XT Index Primer 2 (S5xx), and 12.5µl of NEBNext High-Fidelity 2X PCR Master Mix, with thermocycling at 95°C for 3 min, followed by 13 cycles of 95°C for 30 s, 55°C for 30 s, and 72°C for 30 s, and a final extension at 72°C for 5 min. The final PCR products were cleaned with Agencourt AMPure XP beads and standardised to the same concentration, purified once more using Agencourt AMPure XP beads, and eluted in 30µl to increase the final DNA concentration. Concentration and quality of the PCR amplicons was evaluated using the Qubit™ dsDNA HS Assay Kit (Thermo Fisher Scientific, Fair Lawn, NJ, USA), and a fragment analyser (Agilent, Agilent Technologies Inc., Santa Clara, CA, USA). Amplicons were sequenced using the Illumina® HiSeq 2500 platform (Illumina, San Diego, CA, USA) to generate 2 x 300 bp paired-end reads. 15% PhiX fragment library was added to increase sample diversity.

Shotgun metagenomic analysis

To maximise the chances of achieving sufficient sequencing depth, cows were selected at random from those whose previous 16SrRNA samples had a DNA content of >5ng/µl after the initial DNA extraction, as measured using the Qubit™ dsDNA HS Assay Kit. Microbial DNA was extracted from a second set of swabs that had been collected parallel to those used in the marker gene analysis. The DNA extraction method was the same, using the PureLink™ Microbiome DNA Kit (Invitrogen, Carlsbad, CA, USA) according to the manufacturer's instructions. Agarose gel electrophoresis was carried out using SYBR green as the nucleic acid stain (Thermo

Fisher Scientific Fair Lawn, NJ, USA) to ensure presence of clear DNA bands. Library preparation was carried out on gDNA samples using the Nextera XT kit (Illumina). gDNA input was quantified using Qubit™ to ensure 1ng of each sample was submitted for tagmentation. Whole samples were used in a limited amplification step (12 cycles) which incorporates the indexes at the priming step. Libraries were cleaned using 0.6x AMPure beads. After elution, the quantity of the library was checked by Qubit™ assay and the quality checked on a DNA high sensitivity Bioanalyser chip. The final libraries were pooled in equimolar ratio and the quantity and quality of the final pool was assessed by Qubit™ and Bioanalyser (Agilent 2100 Bioanalyser, Agilent Technologies Inc, Santa Clara, CA, USA), and subsequently by qPCR using the Illumina Library Quantification Kit from KAPA (KK4854) on a Roche Light Cycler LC480II according to manufacturer's instructions. The final loading concentration of 300 pM was reached by adding 35µl exclusion amplification enzyme mix. The libraries were sequenced on an Illumina HiSeq 4000 platform using sequencing by synthesis (SBS) technology to generate 2 x 150 bp paired-end reads.

Quality control and filtering of shotgun metagenomic sequences

Data files were demultiplexed and converted to FASTQ format using Casava v.1.8.2 (Illumina). FASTQ files were trimmed using option _O3 Cutadapt version 1.2.1 (1) to exclude those matching Illumina adaptor sequences by ≥ 3bp at the 3’ end. The reads were further trimmed to remove low quality bases,

using Sickle version 1.200 with a minimum window quality score of 20. After trimming, reads shorter than 20 bp were removed, and single reads were excluded as length distributions showed they were of poor quality. Host reads were removed following alignment against the host Bos Taurus genome using Bowtie2 v2.2.6 (2): read pairs where one or both reads aligned were removed. The remaining reads in pairs were merged using PEAR v0.9.11 (3) to form a single long read based on overlapping homology. Those that could not be merged in this way were concatenated with an intervening N-base. The resulting sequences underwent taxonomic assignment using Kraken v0.10.6 (4) and results were filtered using a confidence threshold of 0.1. Results were analysed using Linear discriminant analysis effect size (LefSe) (5) to determine the taxa most likely to explain differences between the two classes HtHt and HtIn. The HUMAnN2 search strategy (6) was used to functionally annotate read data and abstracts to show biological pathway abundance and completeness. Finally, reads that did not align to their pangenomes using this strategy were submitted to a protein database (UniRef) for translated searching (6). The gene families identified were further analysed using the MetaCyc database to reconstruct and quantify complete metabolic pathways (7).

Samples were renormalized for sequence depth and terms converted using GO slim (EMBL-EBI, Cambridgeshire, UK) where possible to generate heatmaps representing gene

functions categorised as biological processes, molecular functions, and cellular components.

Genome Wide Association and regional heritability mapping study of foot skin microbiota related traits

Animal sampling and genotyping are described by Sánchez-Molano et al. (8). The phenotypic traits (10 traits) analysed here were three different alpha diversity indices; Chao1, Shannon, Simpson indices, and relative abundances of seven genera; *Porphyromonas* spp., Clostridiales Family XI, *Fastidiosipila* spp., *Peptoclostridium* spp., *Macrococcus* spp., *Treponema* spp., and genera of the family Bacteroidetes.

A genotype call rate threshold of 95%, minor allele frequency (MAF<0.01), Hardy-Weinberg equilibrium (threshold of 1.45E-6 Bonferroni corrected), sample call rate threshold of 95% were applied. 236 out of 242 cows with foot microbiota records were eventually analysed because 6 cows were lost in QC steps. Wald tests using ASReml software package (9) were used to determine statistically significant (*P*-value= 0.05) fixed effects to be included in the analysis as described in Sánchez-Molano et al. (2019). After performing this analysis for all foot microbiota phenotypic traits, concordant models were chosen including farm, parity and season as fixed effects.

The Genomic relationship matrix (GRM) was computed using GEMMA (10) and principal components analysis (PCA) was used to find out any genetic structure of the cow population. This population structure was accounted for in GWA models by

automatically fitting the GRM as part of the polygenic effect, whereas in RHM analysis the first 7 PCs were fitted to account for this structure (RHM analyses failed to converge when the GRM was fitted); further correction for the inflation factor (λ) was applied as described by Amin et al. (11). REACTA (12) was first used to assess the full genomic variance for each trait with a general explanatory analysis. GWA was performed using GEMMA (10) and the following linear mixed model:

$$\mathbf{y=W\alpha + x\beta + u + \varepsilon}$$

where **y** represents the vector of foot microbiota phenotypes, **W** is the incidence matrix for $\boldsymbol{\alpha}$**,** which is the vector of associated fixed effects, **x** is the vector of genotypes which were coded as 0/1/2, $\boldsymbol{\beta}$ is phenotype's regression on genotypes, and **Z** is the design matrix for the vector **u** of random polygenic effects (distributed as MVN(0,V_g**G**), with **G** being the GRM matrix and V_g being the genetic variance), and $\boldsymbol{\varepsilon}$ represents the vector of residual errors (distributed as MVN(0,V_e**I**), with **I** being the identity matrix and V_e being the residual variance). Further λ correction was applied to lower any potential inflation.

The RHM was performed using the following model:

$$\mathbf{y=W\alpha+Xu_{(i)}+\varepsilon}$$

where **y** corresponds to the vector of phenotypes, **W** corresponds to the design matrix for the fixed effects $\boldsymbol{\alpha}$ (including the principal components), **X** is the corresponding design matrices for the effects $\mathbf{u}_{(i)}$ of the corresponding region i (distributed as MVN(0,$Vg_{(i)}\mathbf{G}_{(i)}$), with $Vg_{(i)}$ and $\mathbf{G}_{(i)}$ being

the genomic variance and the GRM corresponding to the SNPs in the ith region, respectively) and ε being the error.

The significance of the region effect was assessed using the likelihood ratio test statistic. A total of 1733 regions were analysed, leading to a genome-wide significant threshold ($P = 0.05$) defined at $P = 2.89E{-}5$ with Bonferroni correction for multiple regions (–log10 (P) = 4.54) and a suggestive threshold (one false positive per genome scan) defined at $P = 5.77E{-}4$ (–log10 (P) = 3.24).

GWA and RHM results were compared to determine common significant/ suggestive regions, and the proportion of variance explained by each region was worked out as a percentage of the total genomic variance.

References

1. Marcel Martin. Cutadapt removes adapter sequences from high-throughput sequencing reads. EMBnet. 2011; 17:5–7.
2. Langmead B, Salzberg SL. Fast gapped-read alignment with Bowtie 2. Nat Methods. 2012;9(4):357–9.
3. Zhang J, Kobert K, Flouri T, Stamatakis A. PEAR: A fast and accurate Illumina Paired-End reAd mergeR. Bioinformatics. 2014;30(5):614–20.
4. Wood DE, Salzberg SL. Kraken: Ultrafast metagenomic sequence classification using exact alignments. Genome Biol. 2014;15(3):R46.
5. Segata N, Izard J, Waldron L, Gevers D, Miropolsky L, Garrett WS, et al. Metagenomic biomarker discovery and explanation. Genome Biol. 2011:R60.
6. Franzosa EA, McIver LJ, Rahnavard G, Thompson LR, Schirmer M, Weingart G, et al. Species-level functional profiling of metagenomes and metatranscriptomes. Nat Methods. 2018;15:962–8.
7. Caspi R, Billington R, Ferrer L, Foerster H, Fulcher CA, Keseler IM, et al. The MetaCyc database of metabolic

pathways and enzymes and the BioCyc collection of pathway/genome databases. Nucleic Acids Res. 2015;44(2):471–80.

8. Sánchez-Molano E, Bay V, Smith RF, Oikonomou G, Banos G. Quantitative Trait Loci Mapping for Lameness Associated Phenotypes in Holstein-Friesian Dairy Cattle. Front Genet. 2019;10:926.

9. Gilmour a R, Gogel BJ, Cullis BR, Thompson R. ASReml user guide release 3.0. VSN International Ltd. 2009. p. 275.

10. Zhou X, Stephens M. Genome-wide efficient mixed-model analysis for association studies. Nat Genet. 2012;44(7):821–4.

11. Amin N, van Duijn CM, Aulchenko YS. A genomic background based method for association analysis in related individuals. PLoS One. 2007;2(12):e1274.

12. Cebamanos L, Gray A, Stewart I, Tenesa A. Regional heritability advanced complex trait analysis for GPU and traditional parallel architectures. Bioinformatics. 2014;30(8):1177–9.

Supplementary Table 1. Classification of cows into groups according to their foot health group and farm of origin. (HtHt: The cows which remained healthy during the study, HtIn: The cows which were healthy at sampling, then developed DD, InIn: The cows which had DD in all checkpoints, InHt: The cows which had DD at initial check point then recovered, Unknown: The cows which could not be followed adequately, died or were sold during the study.)

Farm	***HtHt***	***HtIn***	***InIn***	***InHt***	***Unknown***
1	40	16	14	12	1
2	13	18	14	1	5
3	59	14	30	4	1
Total	112	48	58	17	7

Supplementary Figure 1 Taxonomy displayed by A. Twenty most abundant phyla identified in HtIn compared to HtHt samples; and B. Twenty most abundant genera identified in HtHt compared to HtIn samples (HtHt: The cows which remained healthy during the study, HtIn: The cows which were healthy at sampling, then developed DD)

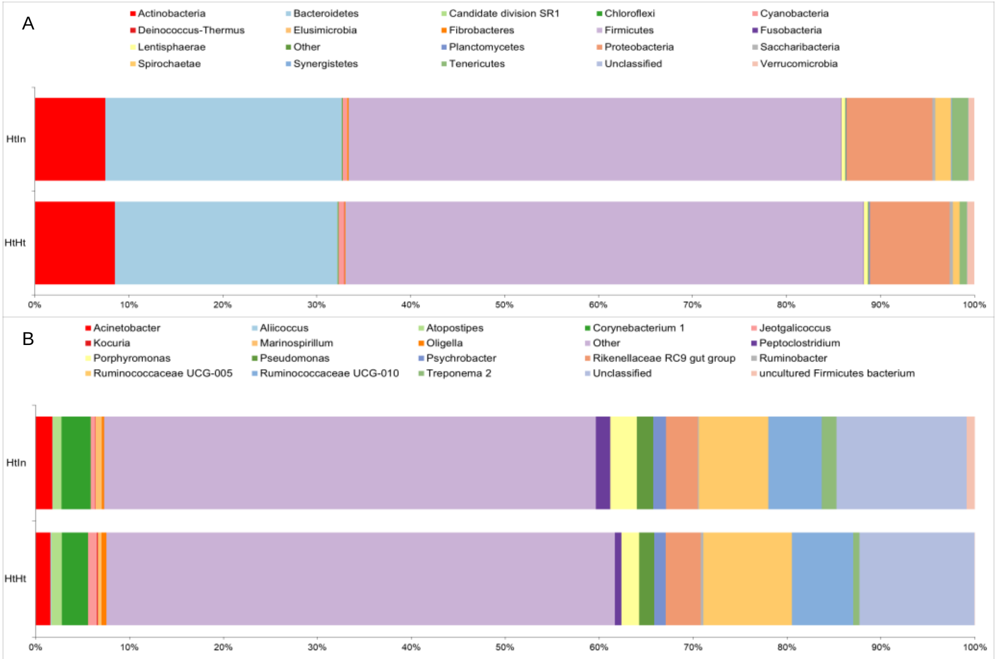

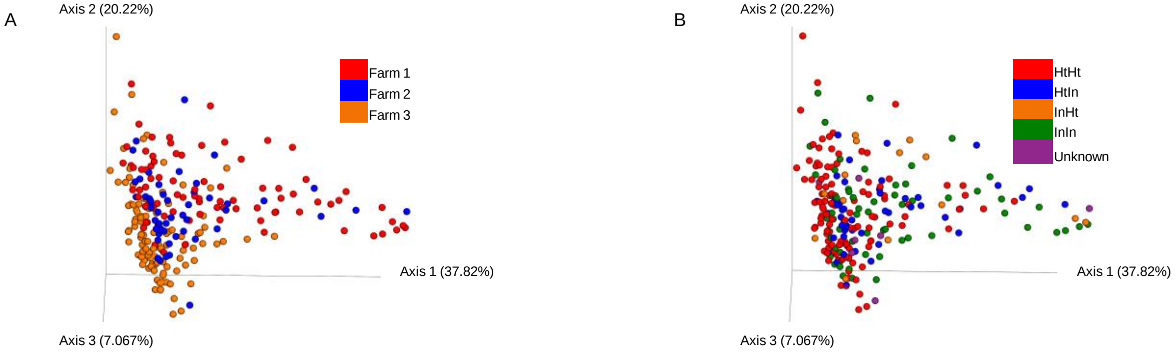

Supplementary Figure 2. Weighted unifrac distances showing beta diversity A. by farm, and B. by foot health group.

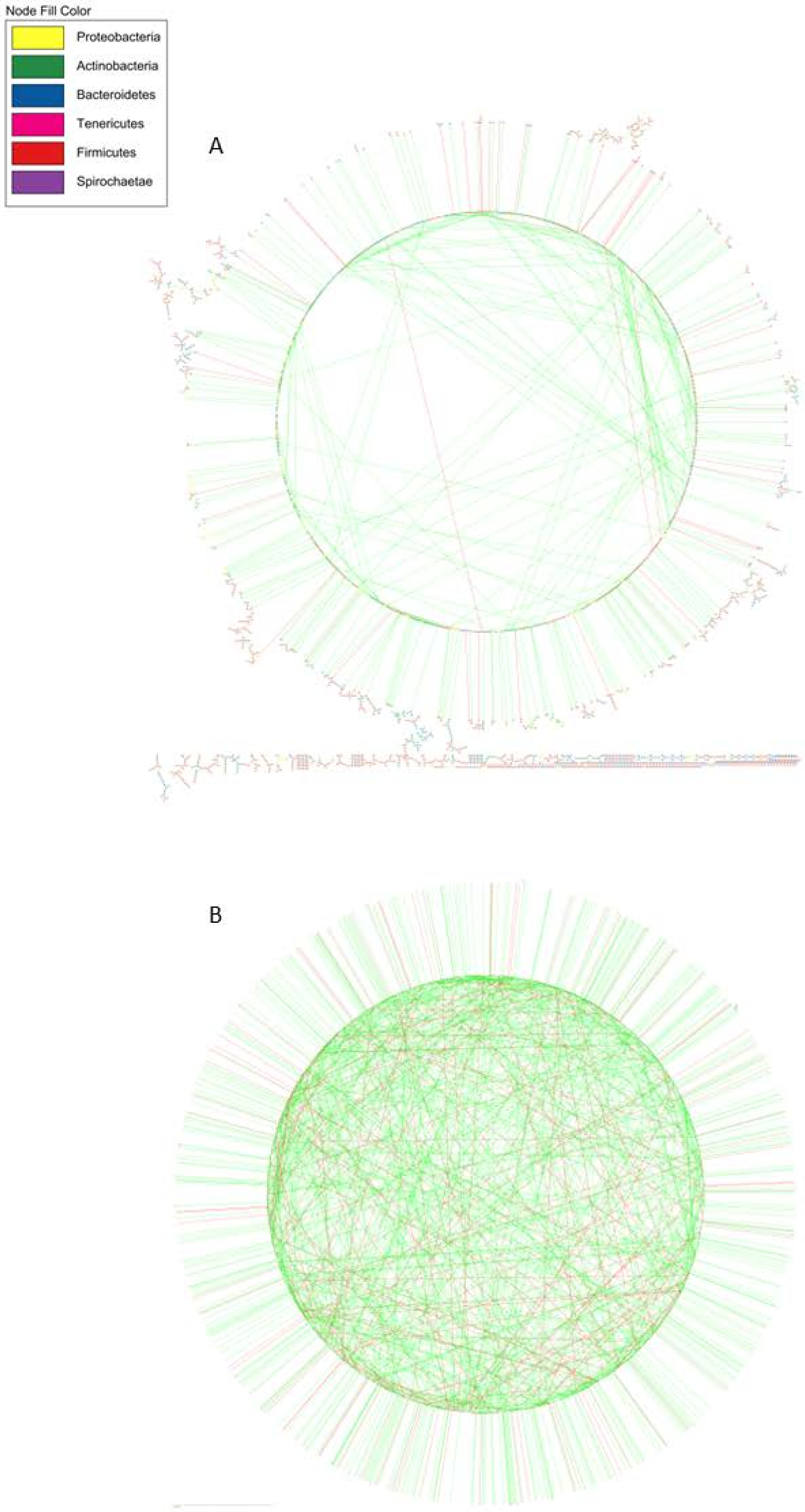
Node Fill Color
Proteobacteria
Actinobacteria
Bacteroidetes
Tenericutes
Firmicutes
Spirochaetae
A
B

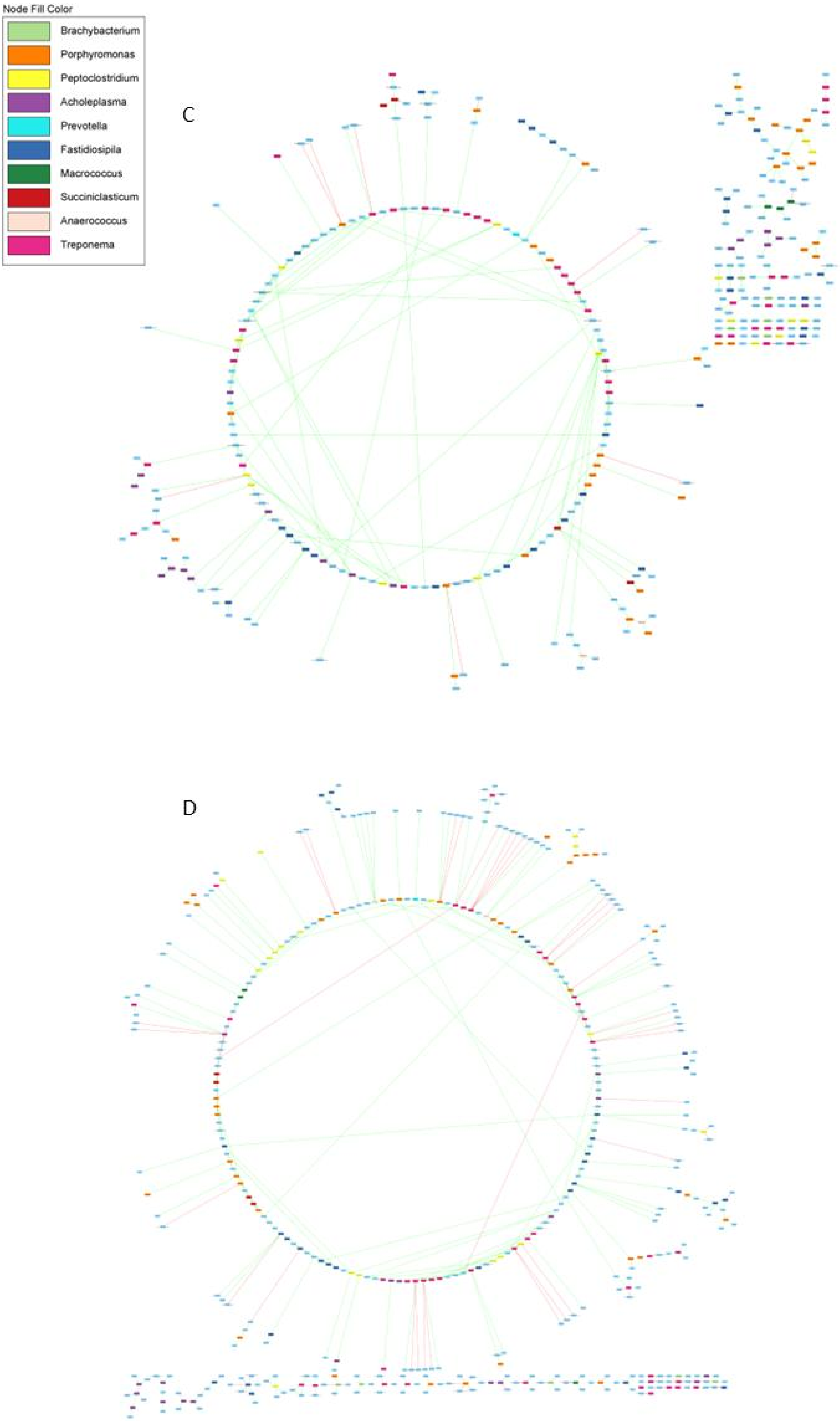
Node Fill Color
Brachybacterium
Porphyromonas
Peptoclostridium
Acholeplasma
Prevotella
Fastidiosipila
Macrococcus
Succiniclasticum
Anaerococcus
Treponema
C
D

Supplementary Table 2. Simple Parameters from Network Analyses for HtHt and HtIn groups carried out using the six phyla that accounted for more than 1% of total OTUs.

**Sample Group**	HtHt n=112	HtIn n=48	Definition
Parameter			
Number of nodes	2339	2802	Nodes represent OTUs
Number of edges	2311	3564	Edges represent correlations (positive or negative) between Nodes (OTUs)
Isolated nodes	18	18	Nodes (OTUs) that are not correlated with any others in the network
Connected components	244	66	Lower = stronger connectivity
Network diameter	51	28	Largest distance between two nodes
Network centralisation	0.003	0.002	How central the most central node is compared to how central all the other nodes are.
Network heterogeneity	0.561	0.503	Reflects the tendency of the network to contain hub nodes: 1 would mean uniformity
Characteristic path length	17.228	9.821	Shorter = stronger connectivity
Average number of neighbours	1.976	2.559	More = stronger connectivity
Clustering coefficient	0.024	0.008	Nodes with <2 neighbours are assumed to have a clustering coefficient of 0
Network density	0.001	0.001	Solely isolated nodes would score 0, cliques would score 1

Supplementary Figure 4. Quantity of functional pathways identified in samples using the Gene Ontology database. A. biological processes, B. cellular components and C. molecular function

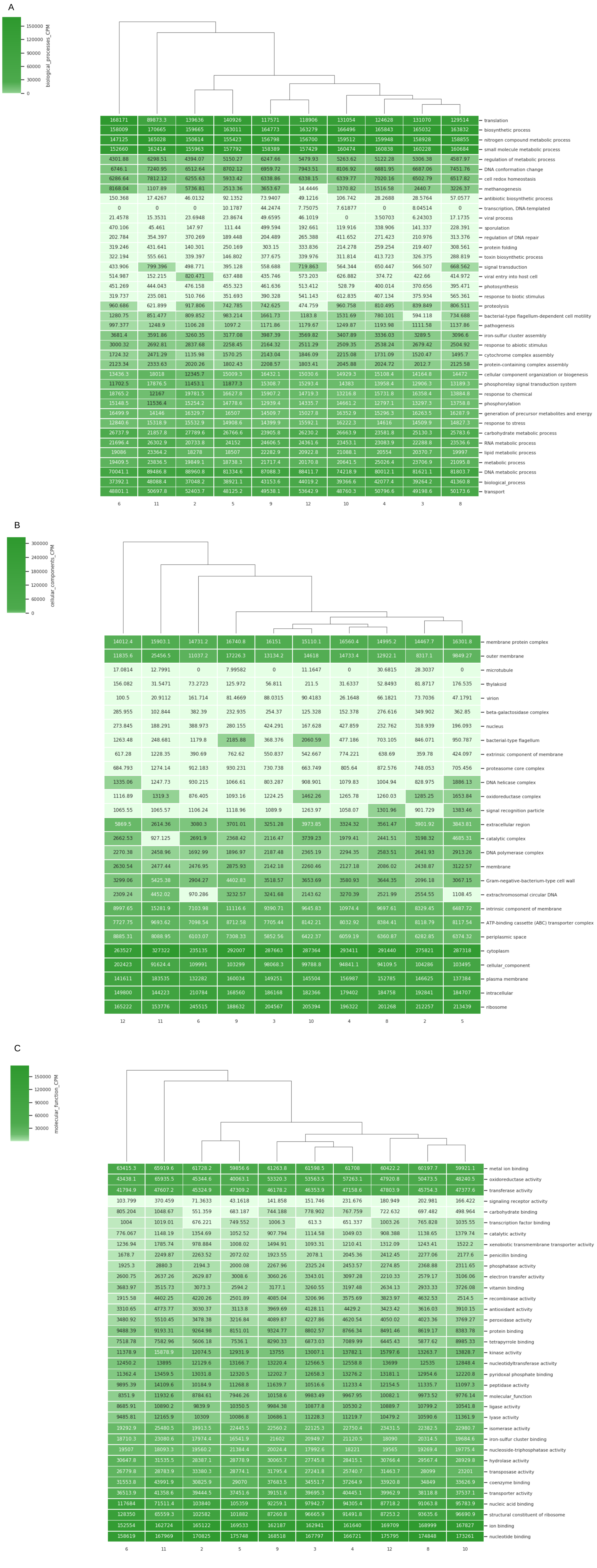

Supplement: Supplementary file 2 — Additional file 1. [file 40168_2022_1440_MOESM1_ESM.pdf]
